# Possible Role of NADPH Oxidase 4 in Angiotensin II-Induced Muscle Wasting in Mice

**DOI:** 10.3389/fphys.2018.00340

**Published:** 2018-04-05

**Authors:** Tomoyasu Kadoguchi, Kazunori Shimada, Hiroshi Koide, Tetsuro Miyazaki, Tomoyuki Shiozawa, Shuhei Takahashi, Tatsuro Aikawa, Shohei Ouchi, Kenichi Kitamura, Yurina Sugita, Al Shahi Hamad, Mitsuhiro Kunimoto, Yayoi Sato-Okabayashi, Koji Akita, Kikuo Isoda, Hiroyuki Daida

**Affiliations:** ^1^Department of Cardiovascular Medicine, Graduate School of Medicine, Juntendo University, Tokyo, Japan; ^2^Sportology Center, Graduate School of Medicine, Juntendo University, Tokyo, Japan; ^3^Laboratory of Molecular and Biochemical Research (Kyodo-ken), Research Support Center, Graduate School of Medicine, Juntendo University, Tokyo, Japan

**Keywords:** angiotensin II, muscle atrophy, oxidative stress, NADPH oxidase 4, Nrf2 signaling

## Abstract

**Background:** Muscle wasting is a debilitating phenotype associated with chronic heart failure (CHF). We have previously demonstrated that angiotensin II (AII) directly induces muscle wasting in mice through the activation of NADPH oxidase (Nox). In this study, we tested the hypothesis that deficiency of NADPH oxidase 4 (Nox4), a major source of oxidative stress, ameliorates AII-induced muscle wasting through the regulation of redox balance.

**Methods and Results:** Nox4 knockout (KO) and wild-type (WT) mice were used. At baseline, there were no differences in physical characteristics between the WT and KO mice. Saline (vehicle, V) or AII was infused via osmotic minipumps for 4 weeks, after which, the WT + AII mice showed significant increases in Nox activity and NOX4 protein compared with the WT + V mice, as well as decreases in body weight, gastrocnemius muscle weight, and myocyte cross-sectional area. These changes were significantly attenuated in the KO + AII mice (27 ± 1 vs. 31 ± 1 g, 385 ± 3 vs. 438 ± 13 mg, and 1,330 ± 30 vs. 2281 ± 150 μm^2^, respectively, all *P* < 0.05). The expression levels of phospho-Akt decreased, whereas those of muscle RING Finger-1 (MuRF-1) and MAFbx/atrogin-1 significantly increased in the WT + AII mice compared with the WT + V mice. Furthermore, nuclear factor erythroid-derived 2-like 2 (Nrf2) and the expression levels of Nrf2-regulated genes significantly decreased in the WT + AII mice compared with the WT + V mice. These changes were significantly attenuated in the KO + AII mice (*P* < 0.05).

**Conclusion:** Nox4 deficiency attenuated AII-induced muscle wasting, partially through the regulation of Nrf2. The Nox4–Nrf2 axis may play an important role in the development of AII-induced muscle wasting.

## Introduction

Muscle wasting is characterized by a loss of muscle mass and strength with decreased physical performance, and its pathogenesis has recently become a subject of great interest (Loncar et al., 2016). Muscle wasting is associated with physical inactivity, poor nutrition, endocrine abnormalities, and chronic heart failure (CHF). It directly impacts physical activity and can adversely affects disease prognosis. Thus, investigating the mechanisms by which muscle mass and function are mediated is crucial.

Muscle wasting can result from physical inactivity and can be caused by catabolic steroids such as glucocorticoids, inflammatory cytokines, reactive oxygen species (ROS), and catabolic nutritional states (Glass, 2003; Braun and Gautel, 2011). Imbalances between protein synthesis and degradation underlie both skeletal muscle atrophy and hypertrophy (Kinugawa et al., 2015). Protein degradation is regulated by several catabolic transcription factors, such as the Forkhead box O (FoxO) and nuclear factor kappa B (NF-kB) proteins. FoxO and NF-kB regulate the transcription of atrogenes and E3 ubiquitin ligases, including muscle RING Finger-1 (MuRF-1) and MAFbx/atrogin-1 (atrogin-1). Conversely, protein synthesis is mainly regulated by the phosphoinositide 3-kinase (PI3K)-Akt cascade. This pathway also regulates the degradation pathway mediated by FoxO protein and atrogenes (Braun and Gautel, 2011).

It is well-established that excessive activation of the renin–angiotensin system (RAS) plays a central role in the pathogenesis and progression of CHF (Harada et al., 1999; Basso et al., 2007; Fukushima et al., 2014). Angiotensin II (AII), a main effector molecule of the RAS, plays an important role in this process. Although the underlying mechanisms are complex and have not been well-studied, muscle wasting is known to be associated with elevated level of circulating AII (Brink et al., 1996, 2001; Yoshida and Delafontaine, 2015). We have previously demonstrated that AII can directly induce muscle wasting by reducing the phosphorylation of Akt, a key molecule in protein synthesis, as well as by increasing the expression of MuRF-1 and atrogin-1, which are both key molecules in protein degradation (Kadoguchi et al., 2015). Additionally, AII activates NADPH oxidase (Nox) and increases Nox-derived ROS activities through their receptors (Griendling et al., 1994; Somanna et al., 2016). We therefore, hypothesized that Nox activation is associated with AII-induced muscle wasting. The purpose of this study was to investigate the effect of NADPH oxidase 4 (Nox4) deficiency on AII-induced muscle wasting and to explore the molecular mechanisms involved.

## Materials and methods

### Animals

To determine the potential involvement of Nox4 in AII-induced muscle wasting, littermate male C57BL/6J [wild-type (WT)] and Nox4-exon 4 knockout (KO) mice (B6.129-Nox4tm1Kkr/J, Jackson Laboratory, Bar Harbor, ME, USA), aged 8–12 weeks, were housed in an animal room under controlled conditions with a 12 h−12 h light–dark cycle. The animals were all individually identified using numeric codes. All the experimental and animal care procedures were approved by our institution's Animal Research Committee and conformed with the guidelines for the Care and Use of Laboratory Animals at Juntendo University Graduate School of Medicine.

### AII-infusion model

AII (Sigma, St. Louis, MO, USA) was continuously infused subcutaneously at 1000 ng kg^−1^ min^−1^ using an osmotic minipump (Alzet model 2004; Alzet Corporation, Palo Alto, CA, USA) for 4 weeks. Saline was used as vehicle (V). To address potential differences caused by food intake, all mice received identical diets, as previously described (Brink et al., 2001). Within each strain, the mice were randomly assigned to either V or AII infusion, which resulted in four experimental groups: WT + V, KO + V, WT + AII, and KO + AII.

### NADPH oxidase activity

Nox activity was measured in homogenates isolated from the gastrocnemius muscle using a lucigenin assay after the addition of NADPH (300 μmol/L) as previously described (Yokota et al., 2009; Takada et al., 2013; Fukushima et al., 2014; Suga et al., 2014).

### Skeletal muscle histology and immunohistochemistry

Changes in the morphology of the gastrocnemius muscle were examined using tissue samples fixed in 4% paraformaldehyde and embedded paraffin. Muscle sections of 8 μm thick were stained with hematoxylin and eosin (HE). The nuclei were counterstained using Mayer's hematoxylin (Muto Chemical Co., Tokyo, Japan). Morphological analysis of the myocyte cross-sectional area was performed in at least 50 cells from each mouse. All images were digitized using a microscope (Olympus BX53; Olympus Optical, Tokyo, Japan) equipped with a high-resolution camera (Olympus DP22).

### RNA isolation, quantitative PCR, and real-time PCR

Total RNA from the gastrocnemius muscle tissue was isolated using PureLink RNA lysis buffer (Invitrogen, Carlsbad, CA, USA). Contaminated genomic DNA was digested with DNase I treatment (Invitrogen) at room temperature for 15 min. Complementary DNA was prepared from the total RNA using reverse transcriptase, according to the manufacturer's instructions (High Capacity cDNA Reverse Transcription Kit; Applied Biosystems, Foster City, CA, USA). Quantitative mRNA expression was assessed by real-time PCR using the Power SYBR Green PCR Master Mix (Applied Biosystems) and target gene-specific primers. All the primers are listed in the supplemental file (Table S1). Samples were analyzed using the 7500 Real-Time PCR system, and the data were analyzed using 7500 Software (Applied Biosystems). Relative expression levels of the WT + V target mRNA group were compared with those of the controls.

### Immunoblotting analysis

Immunoblotting was performed as previously described (Ohta et al., 2011; Inoue et al., 2012; Kadoguchi et al., 2015). Gastrocnemius muscle tissue samples were homogenized in a 1X cell lysis buffer (Cell Signaling, Danvers, MA, USA), supplemented with a 1X complete protease inhibitor cocktail (Roche, Basel, Switzerland) and 1 mmol/L phenylmethylsulfonyl fluoride. After homogenization and centrifugation at 15,000 × g for 10 min at 4°C, the supernatants were collected. Protein aliquots were extracted for total protein assay (Pierce BCA, Rockford, IL, USA). The remaining lysate (20 μg) was added onto a 4–20% gradient or AnykD gels (Bio-Rad, Hercules, CA, USA) and electrophoretically separated with SDS-PAGE using a running buffer. The solution was then transferred to a polyvinylidene fluoride membrane (Bio-Rad) by electroblotting at 100 V using a transfer buffer for 2 h. The membranes were blocked in Tris-buffered saline with 0.1% Tween-20 (TBST) in 5% non-fat dry milk and incubated overnight at 4°C with primary antibodies (dilution 1:1,000) against phosphorylated forms of Akt, Akt itself, phospho-nuclear factor kappa B (p-NF-kB), NF-kB, phospho-p38 mitogen-activated protein kinase (MAPK), p38MAPK (Cell Signaling), MuRF-1, atrogin-1, Nox4, 3-nitrotyrosine, nuclear factor erythroid-derived 2-like 2 (Nrf2), Keap-1, and Histone H3 (Abcam, Cambridge, MA). After washing-three times in TBST buffer, the membranes were incubated with secondary antibodies and conjugated with horseradish peroxidase (dilution 1:5,000; Santa Cruz Biotechnology). They were washed again in TBST and incubated with the chemiluminescence detection reagent in the Amersham ECL Western Blotting Analysis System (GE Healthcare, Chalfont St Giles, UK) for enhanced chemiluminescence. Equal loading of the protein was verified by immunoblotting with glyceraldehyde-3-phosphate dehydrogenase (Cell Signaling), which has been often used as an internal control for western blotting (Reisz-Porszasz et al., 2003; Acharyya et al., 2004). The Proteins were quantified (band × volume) using the LAS-3000 Imaging System (FUJIFILM, Kanagawa, Japan).

### Nuclear fractionation

Nuclear and cytosolic fractions were prepared using a Pierce NE-PER nuclear extraction kit (Pierce, Rockford, IL, USA). Briefly, 50 mg of frozen gastrocnemius muscle tissue was homogenized in CER-I buffer containing protease and phosphatase inhibitors (Pierce). Cytosolic fractions were prepared according to the manufacturer's instructions; pellets containing nuclei were washed four times in PBS to remove cytosolic-contaminating proteins, after which the nuclear proteins were extracted in NER buffer supplemented with inhibitors.

### Statistical analysis

Results are expressed as mean ± SE. For multiple comparisons, two-way ANOVA followed by the Tukey's test was used. *P*-value < 0.05 were considered statistically significant.

## Results

### AII-induced muscle wasting

**Figure 1:**
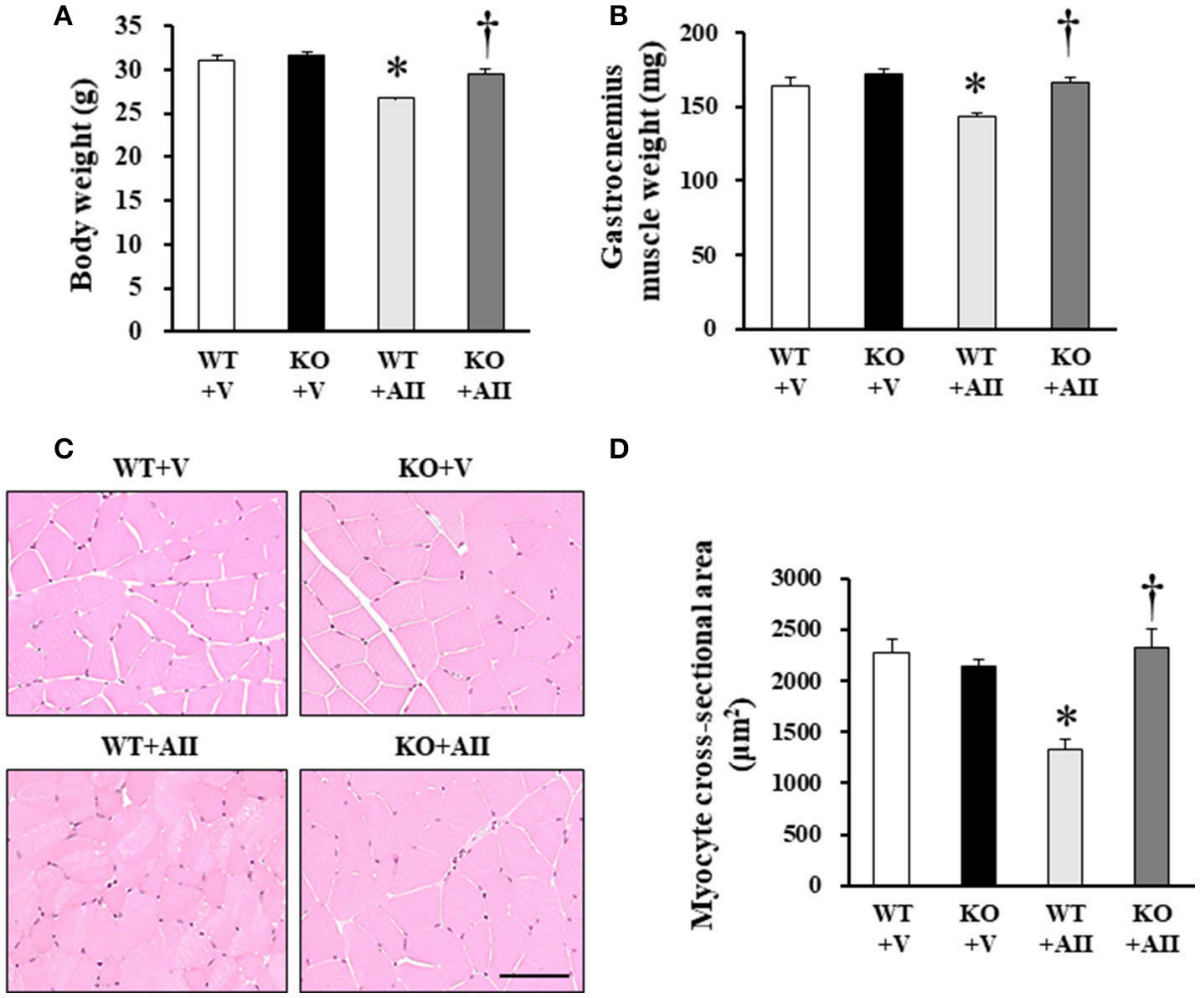
Nox4 deficiency attenuated AII-induced muscle wasting. **(A,B)** Weights of body and lower limbs skeletal muscles, and myocyte cross-sectional area from WT + V, KO + V, WT + AII, and KO + AII mice after 4 weeks (*n* = 7–8 for each group). **(C)** Representative high-power photomicrographs of skeletal muscle tissue sections stained with hematoxylin and -eosin (HE) from four groups of mice. **(D)** Summary data of myocyte cross-sectional areas (*n* = 4 for each group). Scale bar, 100 μm. Data are expressed as mean ± SE. *P < 0.05 vs. WT + V. ^†^*P* < 0.05 vs. WT + AII. WT, wild-type; KO, knockout; V, vehicle; AII, angiotensin II.

No differences were found in the physical characteristics of the WT and KO mice at baseline, but 4 weeks after AII infusion, the gastrocnemius muscle of the WT + AII mice were significantly smaller than those of the WT + V mice. These changes were attenuated in the KO + AII mice (Figures 1A,B). Figure 1C shows representative images of muscle tissue stained with HE. The myocyte cross-sectional area was significantly smaller in the WT + AII than in the WT + V mice, but it was restored in the KO + AII mice (all *P*-values < 0.05) (Figure 1D). An AII-induced increase in heart weight and heart/body weight were not improved in Nox4 KO mice (Table S2), indicating that the progression of AII-induced muscle wasting was independent of cardiac hypertrophy.

### Nox-derived ROS and oxidative stress markers in skeletal muscle

**Figure 2:**
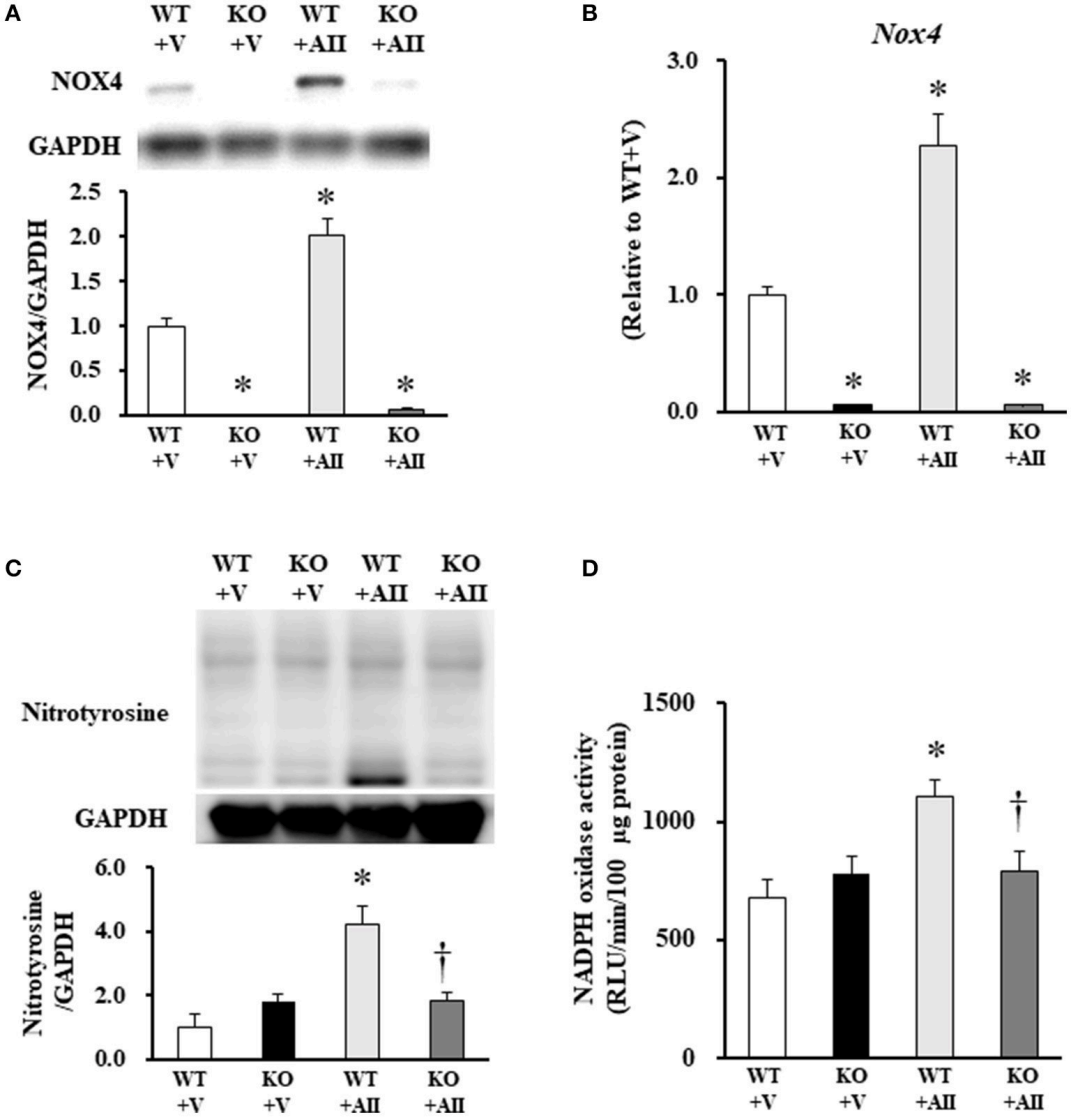
Nox4 deficiency inhibited AII-induced increases in Nox4, NADPH oxidase activity and nitrotyrosine. **(A)** Representative band and summery of the quantitative analysis of NOX4 protein expression level (*n* = 3 for each group), **(B)** mRNA expression level of Nox4 (*n* = 4−6 for each group), **(C)** protein expression level of nitrotyrosine (*n* = 3 for each group), and **(D)** NADPH oxidase activity (*n* = 3−5 for each group) from the four groups of mice. Data are expressed as mean ± SE. *P < 0.05 vs. WT + V. ^†^*P* < 0.05 vs. WT + AII. WT, wild-type; KO, knockout; V, vehicle; AII, angiotensin II. The original images of Figures 2A, C are shown in Figure S2.

NOX4 protein level was increased in muscle sections from the WT + AII mice compared with those from the WT + V mice (*P* < 0.05) (Figure 2A). Similarly, mRNA expression levels of Nox4 were significantly increased in the WT + AII mice compared with the WT + V mice (*P* < 0.05) (Figure 2B). As expected, expression levels of NOX4 protein and mRNA were negligible in the KO mice (Figures 2A,B). The level of nitrotyrosine, a major oxidative stress marker, significantly increased in the WT + AII compared with the WT + V mice, but not in the KO + AII mice (*P* < 0.05) (Figure 2C). Nox activity measured with lucigenin chemiluminescence in muscle homogenates also significantly increased in the WT + AII mice compared with the WT + V mice, but not in KO + AII mice (*P* < 0.05) (Figure 2D).

### Protein synthesis and degradation markers in skeletal muscle

**Figure 3:**
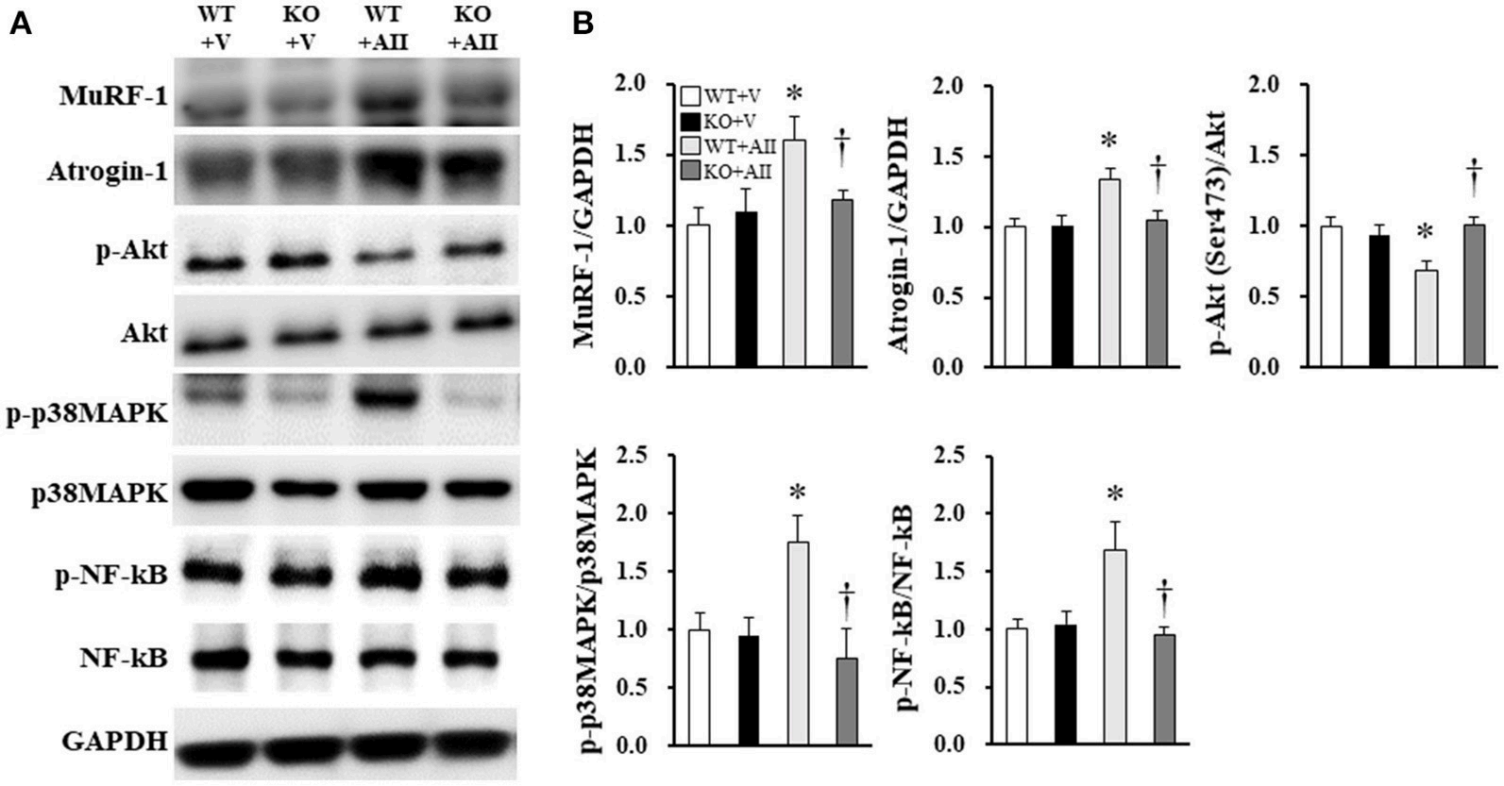
Nox4 deficiency improved the AII-induced increase in protein degradation and decrease in protein synthesis. **(A)** Representative band and **(B)** summary of the quantitative analysis of protein expressions of p-Akt (Ser473), MuRF-1, atrogin-1, p-p38MAPK (Thr180/Tyr182), and p-NF-kβ (Ser536) (*n* = 6 for each group). Data are expressed as mean ± SE. *P < 0.05 vs. WT + V. ^†^*P* < 0.05 vs. WT + AII. WT, wild-type; KO, knockout; V, vehicle; AII, angiotensin II; MuRF-1, Muscle RING Finger-1; atrogin-1, muscle atrophy F-box; p38MAPK, p38 mitogen-activated protein kinase; NF-kβ, nuclear factor kappa-β. The original images of Figure 3A are shown in Figure S2.

Figure 3A shows the immunoblotting analysis for each marker. Akt phosphorylation of Ser473, a key molecule of protein synthesis, decreased in the WT + AII mice compared with the WT + V mice; this change was significantly lower in the KO + AII mice (*P* < 0.05). MuRF-1, atrogin-1, p38MAPK phosphorylation of Thr180/Tyr182, and NF-kβ phosphorylation of Ser536, which are key molecules in protein degradation, significantly increased in the WT + AII mice compared with the WT + V mice. These changes were significantly lower in the KO + AII mice (*P* < 0.05) (Figure 3B).

### Inflammatory cytokines in skeletal muscle

**Figure 4:**
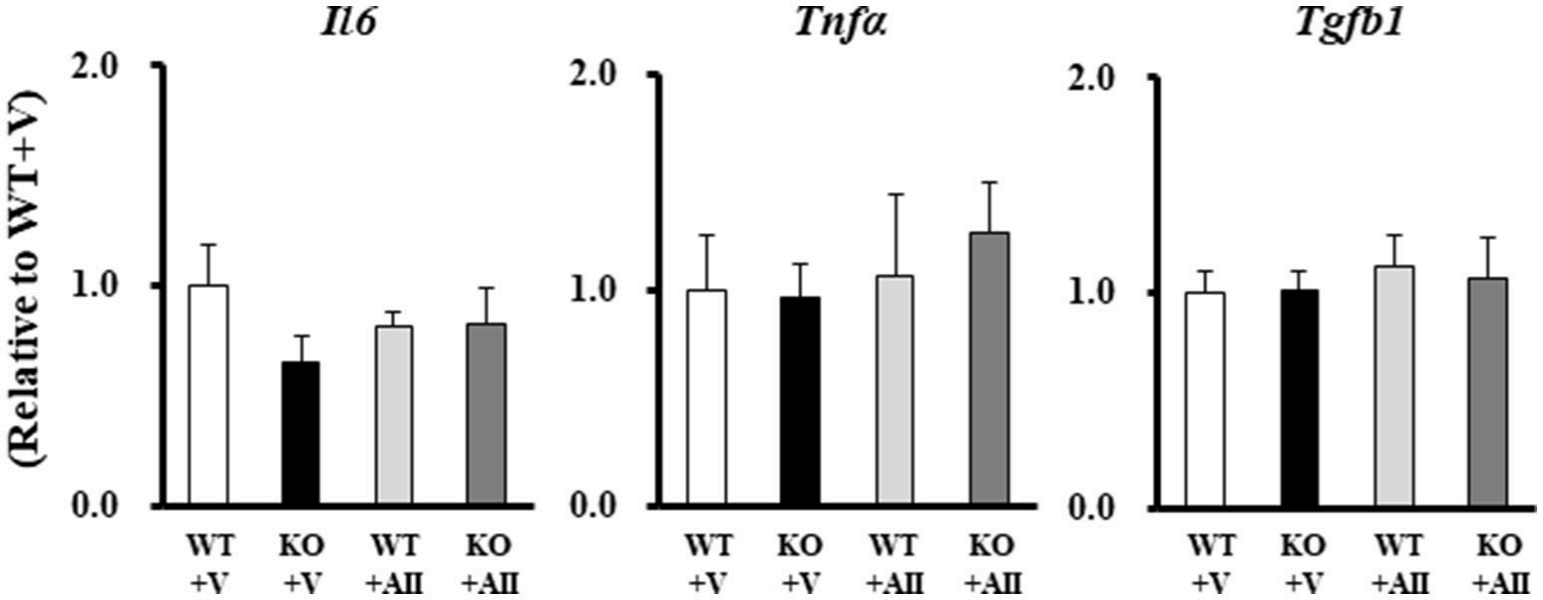
Gene expression levels of inflammatory cytokines in the Nox4 knockout mice. Data are expressed as mean ± SE (*n* = 4–6 for each group). WT, wild-type; KO, knockout; V, vehicle; AII, angiotensin II; TNF-α, tumor necrosis factor-α; IL-6, interleukin-6; TGF-β, transforming growth factor-β.

No group differences were found for gene expression levels in skeletal muscle of the major inflammatory cytokines, tumor necrosis factor-α (TNF-α), interleukin-6 (IL-6), and transforming growth factor-β (TGF-β) (Figure 4).

### Nrf2 and Nrf2-regulated genes in skeletal muscle

**Figure 5:**
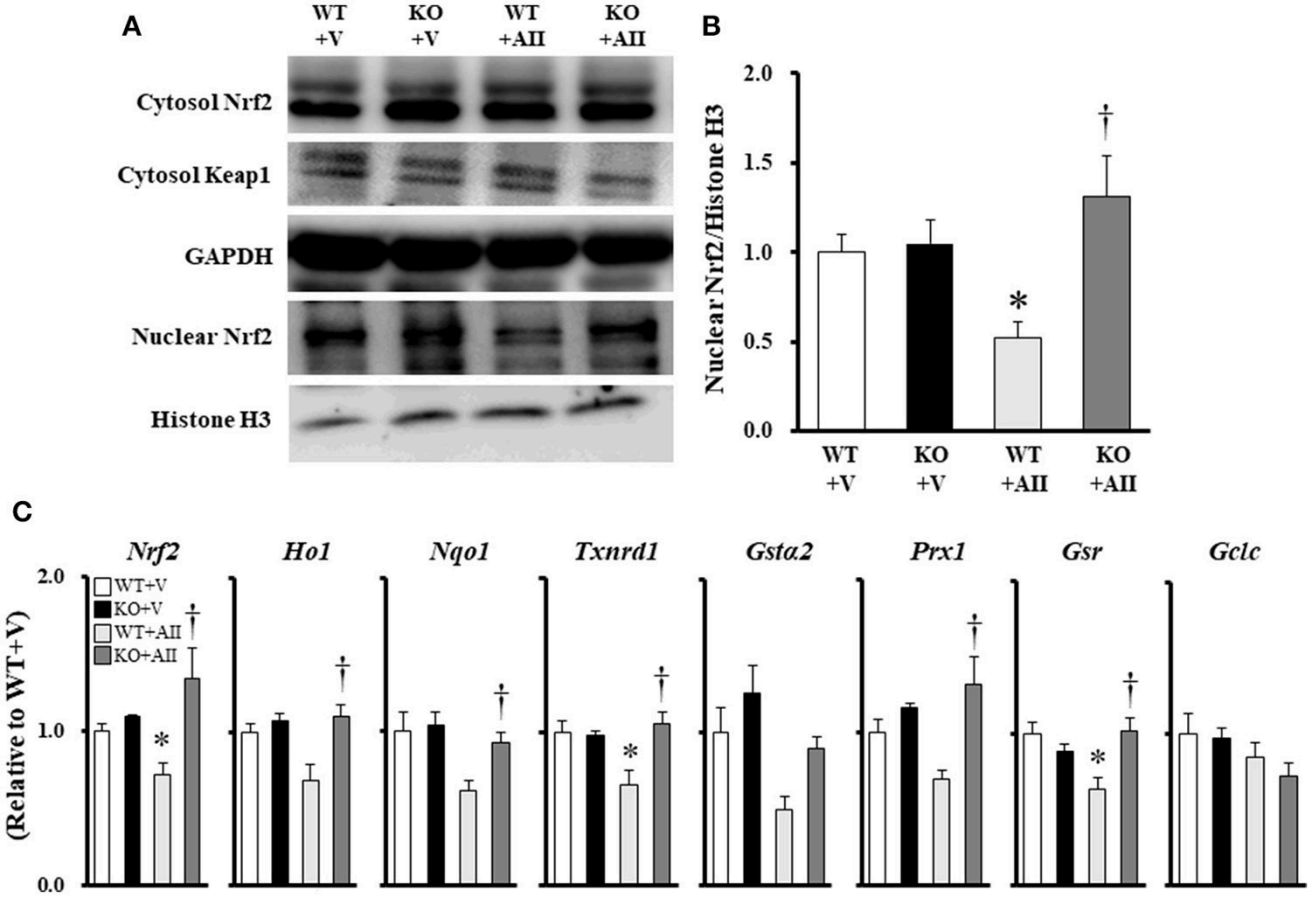
Nox4 deficiency regulates AII-induced decrease in Nrf2 signaling. **(A)** Representative band, and **(B)** summary of the quantitative analysis of the protein expressions of nuclear Nrf2 (*n* = 6 for each group). **(C)** Expression of Nrf2-regulated genes (*n* = 4–6 for each group). Data are expressed as mean ± SE. *P < 0.05 vs. WT + V. ^†^*P* < 0.05 vs. WT + AII. WT, wild-type; KO, knockout; V, vehicle; AII, angiotensin II. Nrf2, nuclear factor erythroid-derived 2-like 2; Keap1, Kelch-like ECH-associated protein 1; HO1, heme oxygenase-1; NQO1, NADPH quinone oxidoreductase 1; Txnrd1, thioredoxin reductase 1; GSTα2, glutathione S-transferase α2; Prx1, peroxiredoxin 1; GSR, glutathione reductase; GCLC, glutamate–cysteine ligase catalytic subunit. The original images of Figure 5A are shown in Figure S3.

Figure 5A shows the immunoblotting analysis for each marker. No differences were found the among groups in the protein expression levels of Nrf2 and Keap1 in the cytosol fraction. However, Nrf2 in the nuclear fraction of the WT + AII mice was significantly lower than that in the WT + V mice (Figure 5B). Furthermore, gene expression levels of Nrf2, thioredoxin reductase 1 (Txnrd1), and glutathione reductase (Gsr) were significantly lower in the WT + AII mice than in the WT + V mice. The expression levels were significantly restored in the KO + AII mice (*P* < 0.05). The results suggested tendencies for decreases in the levels of heme oxygenase 1 (HO1) (*P* = 0.07), NADPH dehydrogenase quinone 1 (NQO1) (*P* = 0.09), and glutathione S-transferase α2 (GSTα2) (*P* = 0.08) in the WT + AII mice compared to the WT + V mice. However, there were no differences in Peroxiredoxin 1 (Prx1) or glutamate–cysteine ligase catalytic subunit (GCLC) between the WT + AII and WT + V mice (Figure 5C).

## Discussion

The aims of this study were to determine the effect of Nox4 deficiency on AII-induced muscle wasting in mice and to explore the molecular mechanisms involved. For this purpose, we used Nox4 KO and WT mice. The findings of showed that Nox4 deficiency (1) attenuated the decrease of body, muscle weight and myocyte cross-sectional area induced by AII, (2) restored the balance between the AII-induced decrease in protein synthesis and increase in the protein degradation in skeletal muscle, and (3) attenuated the decrease in Nrf2 signaling in skeletal muscle. Thus, Nox4-dependent ROS activation may be a therapeutic target for AII-induced muscle wasting.

In the present study, Nox4 deficiency restored the decreased Akt phosphorylation (Figure 3). Previous studies have demonstrated that the activation of Nox also reduced Akt phosphorylation at Ser473 in skeletal myocytes (Wei et al., 2006). Wei et al. suggested that this was due to a decrease in insulin receptor substrate 1 (IRS-1) phosphorylation; did not corroborate this conclusion. Hence, Akt phosphorylation may be impaired by a direct effect of Nox-derived ROS or other signaling pathways. It has been demonstrated that many circulating hormones and cytokines are involved in AII-induced muscle wasting (Yoshida and Delafontaine, 2015). For example, glucocorticoids reduce IRS-1-associated PI3K activity in skeletal muscle and increase muscle atrophy (Hu et al., 2009). The glucocorticoid receptor competitively binds to PI3K, reducing its association with IRS-1, and thus leading to a decrease in IGF-1 signaling. Song et al. (2005) showed that a glucocorticoid receptor antagonist blunted AII-induced muscle wasting, suggesting that AII indirectly activates muscle breakdown through glucocorticoid signaling. Thus, IGF-1 and glucocorticoid signaling may mediate the results described in this report.

It is well-established that protein degradation is involved in AII-induced muscle wasting and may be prevented by the presence of antioxidants in skeletal myocytes (Russell et al., 2007). Furthermore, previous studies have demonstrated that muscle wasting and 20S proteasome activity were attenuated in p47^*phox*^-deficient mice (Semprun-Prieto et al., 2011) suggesting that activation of MuRF-1 and atrogin-1 by ROS may be involved in these processes. Conversely, the inhibition of NF-kB and p38MAPK has been shown to suppress E3 ubiquitin ligase activation (Li et al., 2005; Haegens et al., 2012). Consistent with these results, increased MuRF-1, atrogin-1, p38MAPK, and NF-kβ phosphorylation were all restored in Nox4 KO mice (Figure 3) in the present study. Major inflammatory cytokines, such as TNF-α, IL-6, and TGF-β, play an important role in protein degradation and muscle wasting. We therefore, examined the gene expression levels of these cytokines in skeletal muscle, but found no differences in their expression levels between the AII-infused and Nox4 KO mice (Figure 4). Although the mechanisms of AII-induced muscle wasting are complex, TGF-β may be associated with Nox4 activation (Hecker et al., 2009; Liu et al., 2016). It has been shown that TGF-β stimulation was gradually increased by Nox4 in vascular smooth muscle cells and myofibroblasts, and that these changes were restored by treatment with Nox4 inhibition (Hecker et al., 2009; Liu et al., 2016). Morales et al. showed that TGF-β increased in the diaphragm muscle of AII-infused mice (Morales et al., 2014), but these results were not consistent with our findings, perhaps because of differences in the duration of AII treatment (2 vs. 4 weeks, respectively) and the muscle studied (diaphragm vs. gastrocnemius muscle, respectively). Therefore, in our model, the restoration of AII-induced muscle wasting may have occurred independently of the TGF-β signaling pathway.

Nox4, a major source of ROS, plays an important role in the pathophysiology of CHF and lung injury (Hecker et al., 2009; Kuroda et al., 2010). However, the precise molecular mechanisms underlying Nox4-dependent cellular functions remain unknown. The transcription factor Nrf2 is a master regulator of oxidative stress, playing a central role in the induction of antioxidative and phase II enzymes (Wasserman and Fahl, 1997; Wakabayashi et al., 2010). Nrf2 is constitutively expressed and degraded by its inhibitor, Keap1, allowing a fast response to stress. Keap1 inhibition is terminated through oxidative stress or direct Nrf2-phosphorylation, with Nrf2 translocating to the nucleus and binding to a specific consensus sequence known as the antioxidant response elements. Nox4 is associated with the regulation of Nrf2 in various tissues (Brewer et al., 2011; Khodo et al., 2012; Hecker et al., 2014). These results suggest that Nox4 may be associated with Nrf2 in AII-induced muscle wasting. However, few studies have examined the association between Nox4 and Nrf2 under atrophic conditions. Interestingly, in the present study, decreased mRNA levels of Nrf2, Txnrd1, Gsr, and Nrf2 in the nuclear fraction were restored in the Nox4 KO mice during AII infusion (Figures 5A–C). Although these results remain controversial, they strongly suggest that the Nox4–Nrf2 axis plays an important role in AII-induced muscle wasting.

One limitation of this study was that we were unable to show an interaction between Nox4 and Nrf2 in AII-induced muscle wasting. Furthermore, Nox4 and Nox2 are abundantly expressed in skeletal muscle and cardiomyocytes (Whitehead et al., 2010; Maejima et al., 2011). We also examined the gene expression levels of other Nox components, specifically Nox2, p22^*phox*^, and p47^*phox*^ (Figure S1). Although Nox2 and p22^*phox*^ did not differ among the groups, p47^*phox*^ was significantly higher in the WT + AII, and its expression was not improved in the KO + AII mice. This suggested a lack of association between AII-induced muscle wasting and the other Nox components, including Nox2, even though Nox2 is abundantly expressed in skeletal muscles. However, further research is needed to elucidate the role of Nox2 in AII-induced muscle wasting using Nox2 KO mice as an animal model.

This study demonstrated that AII-induced muscle wasting was mediated by the activation of Nox4 and, at least partially, by downregulation of Nrf2 signaling in skeletal muscles, leading to stimulation of the protein degradation pathways. These findings have important implications for understanding the mechanisms that underlie weight and muscle loss in conditions such as heart failure, in which RAS is activated.

## Author contributions

TK, HK, TM, TS, ST, TA, SO, KK, and YS were responsible for conducting the experiments and analyzing and interpreting the data. TK, AH, KA, YS-O, MK, and KI were involved in drafting the manuscript for publication. KS and HD were responsible for conceiving all experiments and was involved in analyzing the data, preparing them for publication, and drafting the manuscript.

### Conflict of interest statement

The authors declare that the research was conducted in the absence of any commercial or financial relationships that could be construed as a potential conflict of interest.
