# Supplementary figures and images for: Possible Role of NADPH Oxidase 4 in Angiotensin II-Induced Muscle Wasting in Mice
Source: Front Physiol. 2018 Apr 5;9:340. doi: 10.3389/fphys.2018.00340 (PMC5895660; doi:10.3389/fphys.2018.00340)

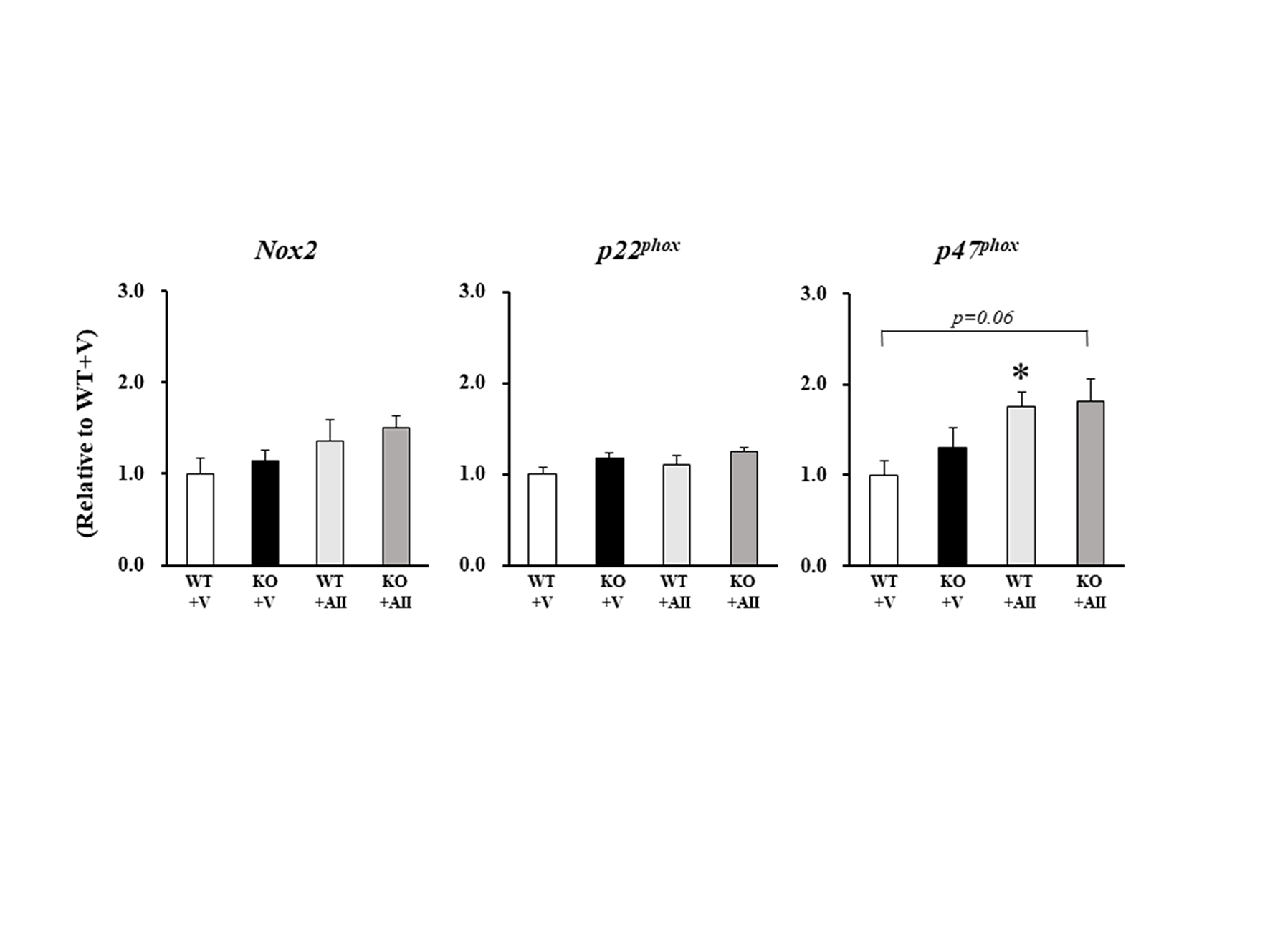

Supplement: Supplementary file 1 [file Image1.TIF]

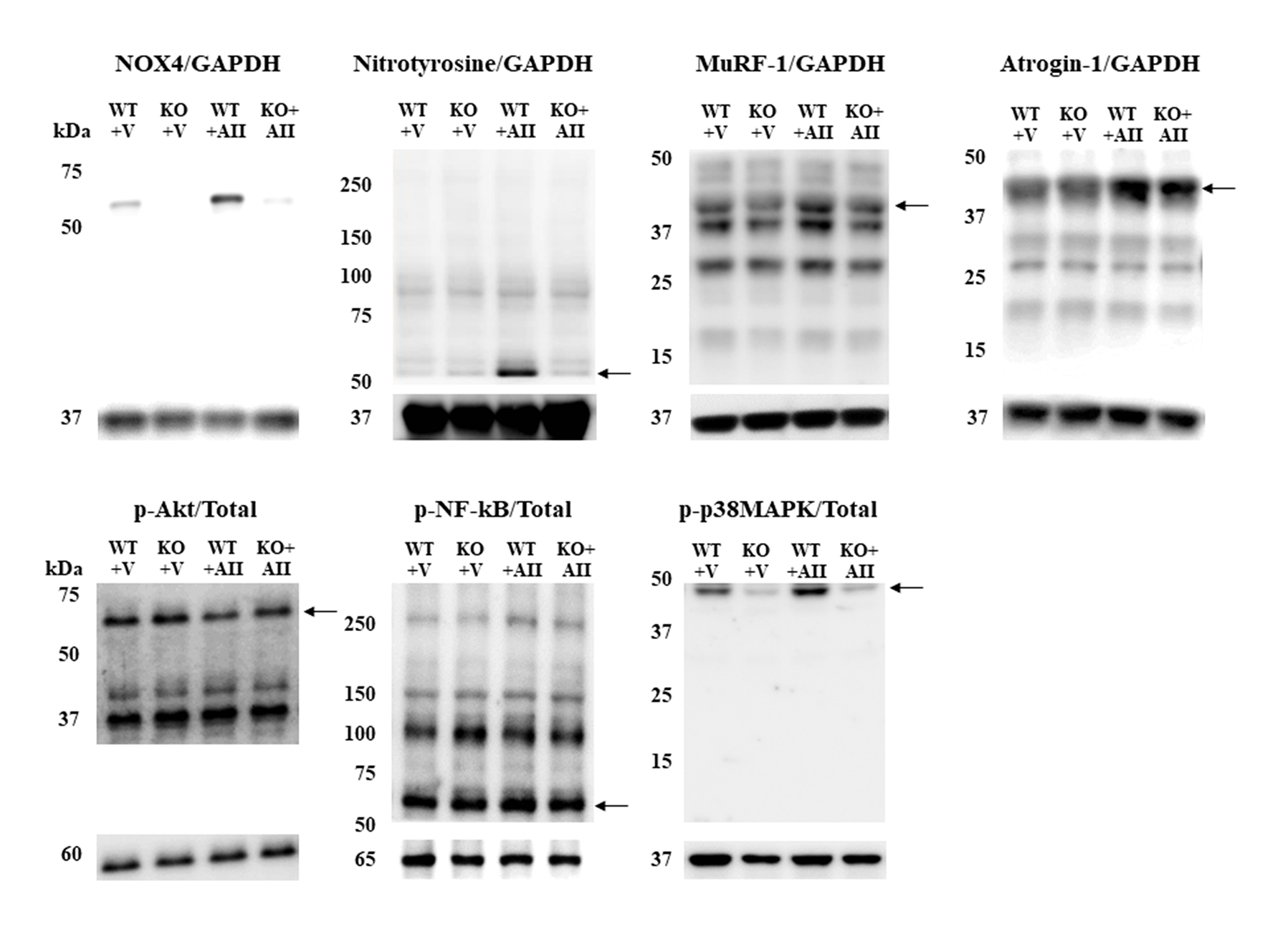

Supplement: Supplementary file 2 [file Image2.TIF]

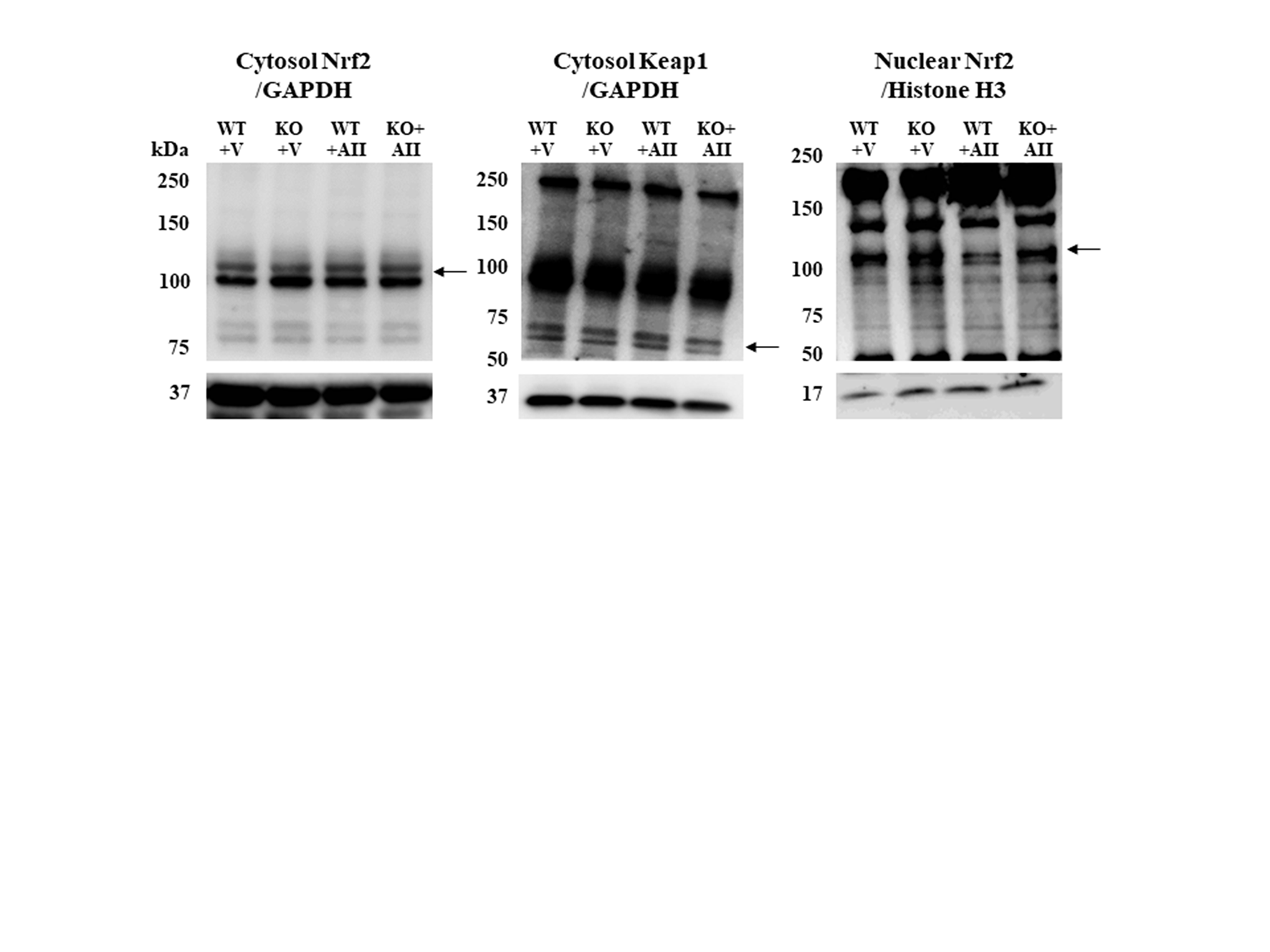

Supplement: Supplementary file 3 [file Image3.TIF]
