# Supplementary material for: Possible Role of NADPH Oxidase 4 in Angiotensin II-Induced Muscle Wasting in Mice
Source: Front Physiol. 2018 Apr 5;9:340. doi: 10.3389/fphys.2018.00340 (PMC5895660; doi:10.3389/fphys.2018.00340)
Supplement: Supplementary file 4 [file Table1.DOCX]

**Supplementary Materials**

**Table S1. Mouse PCR primers for qRT-PCR.**

**Table S2. Animal characteristics in the WT and the Nox4 KO mice.**

Data are expressed as mean ± SE. **P* < 0.05 vs. WT + V. N = 7–8 for each group.

**Figure S1. Gene expression levels of Nox components Nox2, p22*^phox^*, and p47*^phox^*.**

Data are expressed as mean ± SE. **P* < 0.05 vs. WT + V. N = 4-6 for each group.

**Figure S2. Original image of oxidative stress, protein synthesis and degradation markers.**

Original images of NOX4, nitrotyrosine, MuRF-1, atrogin-1, p-Akt (Ser473), total Akt, p-p38MAPK (Thr180/Tyr182), p-p38MAPK, p-NF-kβ (Ser536), and total NF-kβ.

**Figure S3. Original image of Nrf2 and Keap1.**

Original images of Nrf2 and Keap1 in the cytosol and nuclear fraction.

**Table S1.** Mouse PCR primers for qRT-PCR.

| Nox4_Forward | TCCCTAGCAGGAGAACAAGAA |
| --- | --- |
| Nox4_Reverse | TTGCTGCATTCAGTTCAAGG |
| Nox2_Forward | ACTCCTTGGGTCAGCACTGG |
| Nox2_Reverse | GTTCCTGTCCAGTTGTCTTCG |
| p22*^phox^*_Forward | ATGGAGCGATGGTTGTCGG |
| p22*^phox^*_Reverse | AATGGGAGTCCACTGCTCAC |
| p47*^phox^*_Forward | TTCATCCCCAGCCAGCACTA |
| p47*^phox^*_Reverse | TCCTTCAGCATTTTATGGAACTCGT |
| MuRF-1_Forward | GGGAACGACCGAGTTCAGACTATC |
| MuRF-1_Reverse | CCTTCACCTGGTGGCTGTTTTC |
| Atrogin-1_Forward | GCTTGTGCGATGTTACCCAAGAA |
| Atrogin-1_Reverse | GAAAGTGAGACGGAGCAGCTCT |
| Nrf2_Forward | CCATATTCCATTCCCTGTCG |
| Nrf2_Reverse | TAAGTGGCCCAAGTCTTGCT |
| HO1_Forward | CCCACCAAGTTCAAACAGCTC |
| HO1_Reverse | AGGAAGGCGGTCTTAGCC |
| NQO1_Forward | AGGATGGGAGGTACTCGAATC |
| NQO1_Reverse | TGCTAGAGATGACTCGGAAGG |
| GCLC_Forward | CGGGCCGATGGTCACGTCGCC |
| GCLC_Reverse | GCAGAGTGCTGAGTCACGGTGAG |
| Prx1_Forward | GATCCCAAGCGCACCATT |
| Prx1_Reverse | TAATAAAAAGGCCCCTGAAAGAGAT |
| GSTα2_Forward | GCTTGATGCCAGCCTTCTG |
| GSTα2_Reverse | GGCTGCTGATTCTGCTCTTGA |
| Txnrd1_Forward | GATGCACCAGGCAGCTTTG |
| Txnrd1_Reverse | TCTTCGACTTTCCAGCCATAGT |
| TNF-α_Forward | TCCCAGGTTCTCTTCAAGGGA |
| TNF-α_Reverse | GGTGAGGAGCACGTAGTCGG |
| IL-6_Forward | ACAACCACGGCCTTCCCTACTT |
| IL-6_Reverse | CACGATTTCCCAGAGAACATGTG |
| TGF-β1_Forward | TTCCTGGCCTTACCTTGG |
| TGF-β1_Reverse | CCTGTATTCCGTCTCCTT |
| GAPDH_Forward | GTCATTGAGAGCAATGCCAG |
| GAPDH_Reverse | GTGTTCCTACCCCCAATGTG |

**Table S2. Animal characteristics.**

|  | WT + V | KO + V | WT + AII | KO + AII |
| --- | --- | --- | --- | --- |
| N | 7 | 8 | 7 | 8 |
| Heart weight (mg) | 125.0 ± 3.1 | 139.0 ± 3.7 | 162.3 ± 5.1* | 174.1 ± 6.4* |
| Heart weight/body weight | 4.0 ± 0.1 | 4.4 ± 0.1 | 6.0 ± 0.2* | 5.9 ± 0.2* |

Data are expressed as mean ± SE. V, vehicle; AII, angiotensin II. **P* < 0.05 vs. WT + V.
